# Supplementary material for: Blood monocyte transcriptome and epigenome analyses reveal loci associated with human atherosclerosis
Source: Nat Commun. 2017 Aug 30;8:393. doi: 10.1038/s41467-017-00517-4 (PMC5577184; doi:10.1038/s41467-017-00517-4)
Supplement: Supplementary file 3 — Supplementary Information [file 41467_2017_517_MOESM3_ESM.pdf]

Description of Supplementary Files

File name: Supplementary Information
Description: Supplementary figures, supplementary tables, supplementary methods and supplementary references.

File name: Supplementary Data 1
Description: mRNA expression alterations in LPS stimulated human THP1-monocytes resulting from ARID5B knockdown. †ARID5B TFBS within 20kb of the gene start/stop coordinates, from HMR Conserved Transcription Factor Binding Sites for ARID5B

File name: Peer Review File

A) Carotid plaque score

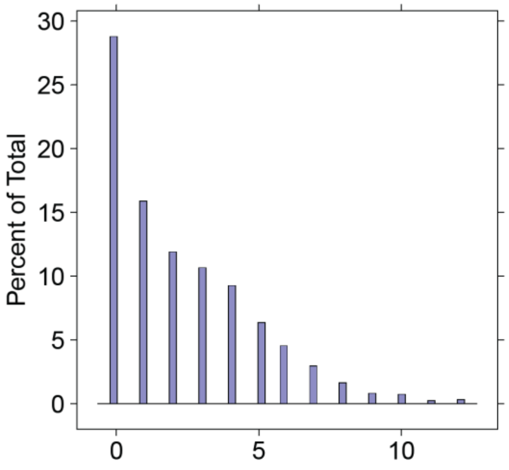

Carotid plaque score

B) Coronary artery calcium score

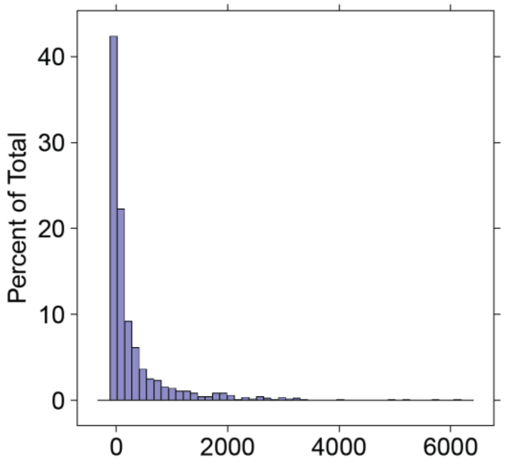

CAC

Supplementary Figure 1. Histograms of subclinical CVD phenotypes

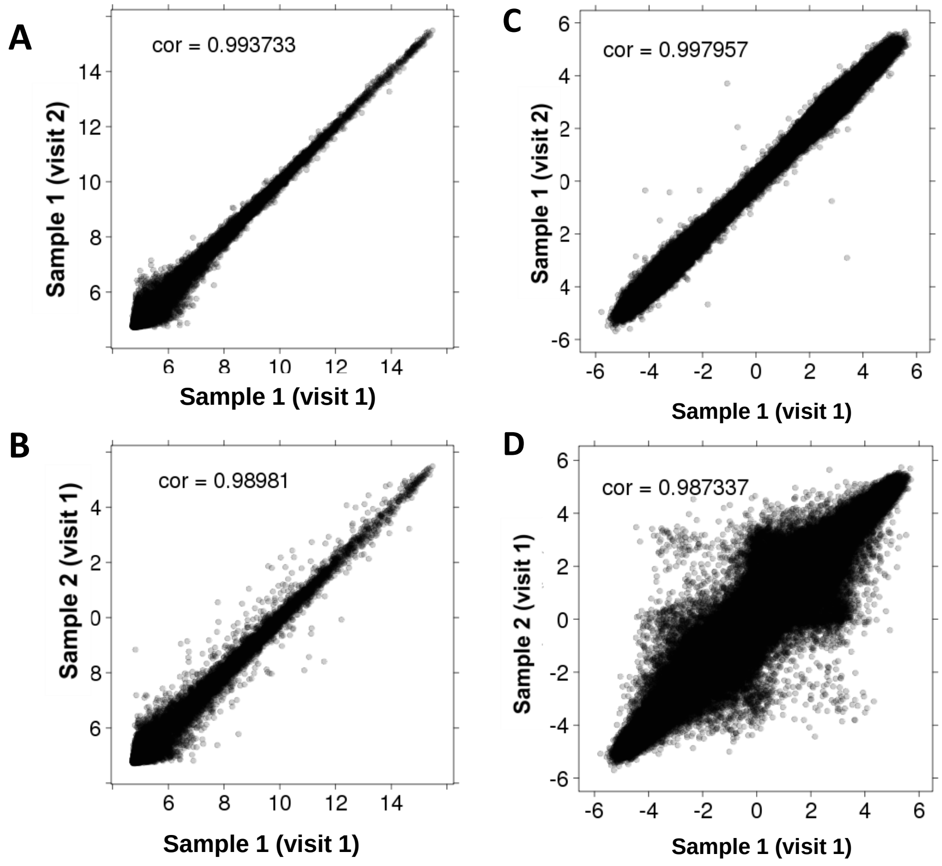

Supplementary Figure 2. Reproducibility of Microarray mRNA Expression and DNA Methylomic Data Overtime. A) Correlation of the repeated measurements of transcriptomic profiles for one subject between two visits 5-month apart; **B)** correlation of transcriptomic profiles between two subjects at visit 1; **C)** Correlation of the repeated measurements of methylomic profiles for one subject between two visits 5-month apart; **D)** correlation of methylomic profiles between two subjects at visit 1.

CD14+ Chromatin states

CD14+ Chromatin mark frequency (%)

	6	5	4	3	2	1
H3K36me3	2	4	12	3	0	72
H3K27ac	5	99	92	6	0	1
H3K4me1	71	71	89	64	0	1
H3K4me3	91	99	8	0	0	0

	6	5	4	3	2	1
Genome %	1	1	2	3	87	7
Nuclear lamina	0	0	0	0	1	0
CpG Island	40	36	0	1	0	1
RefSeq TSS	27	39	1	1	0	0
CD14+ RNAseq	3	7	4	3	0	6
CD14+ DNase	13	30	14	7	0	0

	6	5	4	3	2	1
Chromatin state annotation	Weak promoter	Active promoter	Strong enhancer	Weak/poised enhancer	Heterochromatin/low	Transcribed

Supplementary Figure 3. Chromatin Map of Monocytes. Six chromatin states (top panel) predicted using ChromHMM and four CD14+ histone marks (from BLUEPRINT) are enriched with various genomic features (middle panel); annotation of the six chromatin states is provided (bottom panel).

A

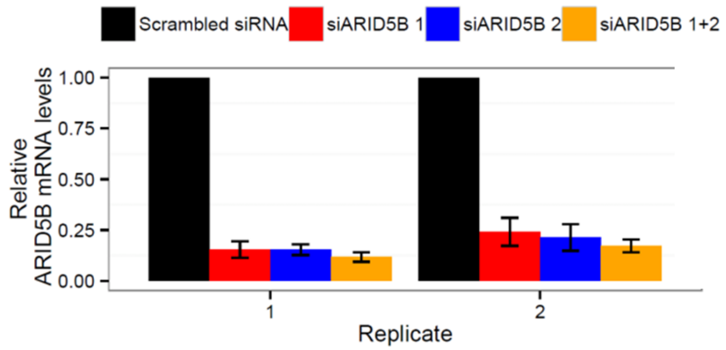

B

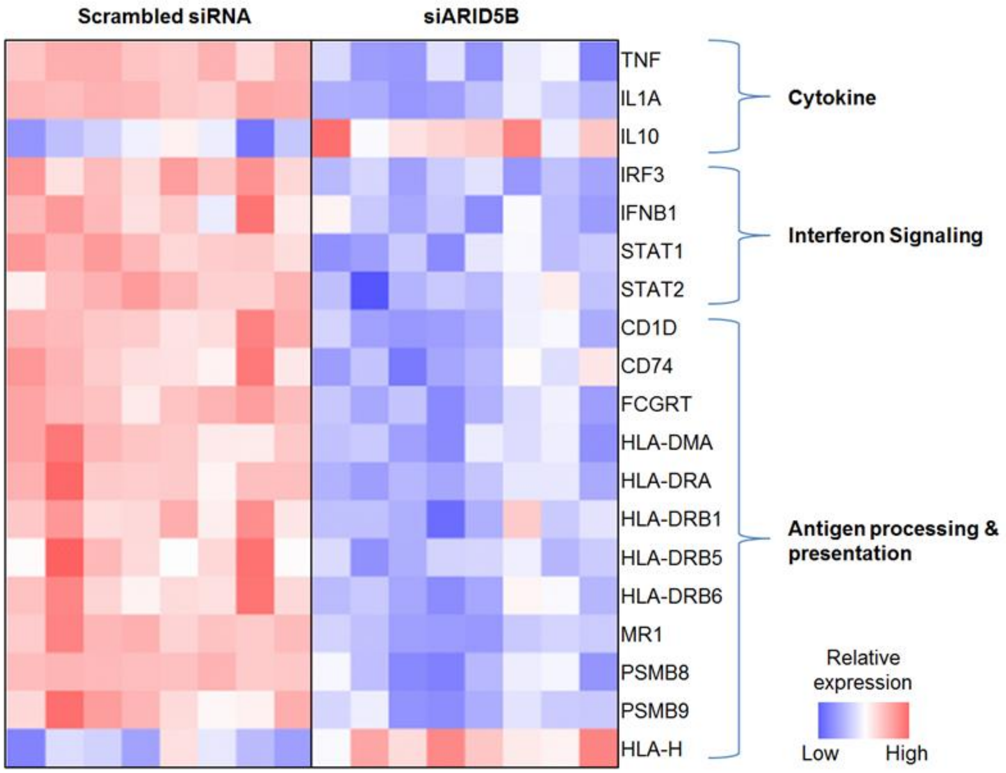

Supplementary Figure 4. siRNA Knockdown of ARID5B alters immune/inflammatory response and lipid metabolism genes. A) Relative ARID5B mRNA expression levels in human THP1-monocytes after treatment with two ARID5B siRNA individually, and in combination. Levels shown are the average (±S.E.M.). After 3 h of LPS treatment (100 ng ml^{-1}), the THP1-monocyte transcriptome in 8 ARID5B knockdown samples compared to 8 control samples (scrambled siRNA) were enriched with inflammatory response genes; **B)** heatmap shows mRNA expression of selected key inflammatory response genes (including cytokine, interferon signaling, and antigen processing and presentation genes) in the 8 scrambled siRNA samples compared to 8 siARID5B samples.

A

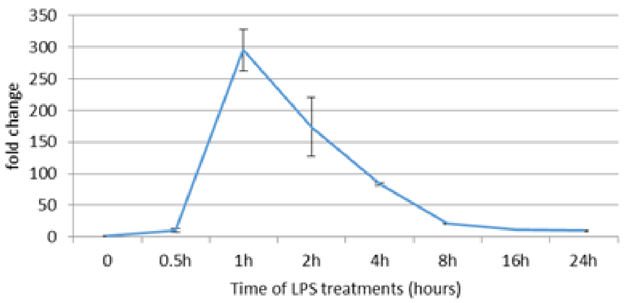

B

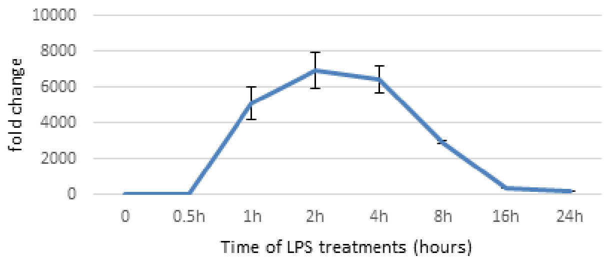

Supplementary Figure 5. Time Course of LPS-induced *TNF* and *IL1A* Gene Expression in Human THP1 monocytes. Gene expression of **(A)** *TNF* and **(B)** *IL1A*, in LPS (100 ng/ml) stimulated THP1 cells

Supplementary Table 1. Monocyte Genes with Expression Associated with Atherosclerosis (FDR < 0.20)

Gene			Carotid Plaque Score (n=1,181)				Coronary Artery Calcium (n=1,208)			
**Gene name**	**Illumina probe id**	**Chr**	**Beta**	**SE**	**P-value**	**FDR**	**Beta**	**SE**	**P-value**	**FDR**
CACNA2D3	ILMN_1754076	3	-0.06	0.02	6.71E-03		-0.44	0.08	2.08E-08	3.19E-04
ARID5B	ILMN_1721626	10	0.12	0.02	6.30E-08	1.08E-03	0.32	0.08	2.47E-05	3.45E-02
PDLIM7	ILMN_1690125	5	0.10	0.02	3.56E-06	3.05E-02	0.31	0.07	8.49E-06	1.95E-02
LILRB4	ILMN_2355953	19	0.07	0.02	1.41E-03		0.32	0.07	6.44E-06	1.95E-02
TSPYL1	ILMN_1779014	6	-0.07	0.02	2.10E-03		-0.34	0.07	3.57E-06	1.95E-02
P2RX4	ILMN_1813846	12	0.04	0.02	4.82E-02		0.31	0.07	8.06E-06	1.95E-02
RSU1	ILMN_2383344	10	-0.02	0.02	0.29		-0.33	0.07	5.29E-06	1.95E-02
FAM168B	ILMN_1677122	2	-0.02	0.02	0.40		-0.32	0.07	1.01E-05	1.95E-02
CANX	ILMN_2401057	5	0.00	0.02	0.86		-0.33	0.07	9.58E-06	1.95E-02
IARS	ILMN_1733956	9	-0.04	0.02	0.07		-0.31	0.07	2.21E-05	3.39E-02
ADM	ILMN_1708934	11	0.02	0.02	0.33		0.32	0.07	2.07E-05	3.39E-02
FAM10A4	ILMN_1726308	13	-0.04	0.02	4.72E-02		-0.29	0.07	3.39E-05	4.31E-02
EIF5A2	ILMN_1735151	3	0.03	0.02	0.16		0.29	0.07	3.64E-05	4.31E-02
EBAG9	ILMN_1791896	8	0.00	0.02	0.99		-0.30	0.07	5.17E-05	0.06
PTPRN2	ILMN_1728844	7	-0.05	0.02	1.34E-02		-0.27	0.07	7.93E-05	0.08
ZNF395	ILMN_1772876	8	-0.02	0.02	0.25		-0.28	0.07	9.50E-05	0.09
IMPDH2	ILMN_1705737	3	-0.01	0.02	0.70		-0.28	0.07	1.00E-04	0.09
TRNT1	ILMN_1764098	3	-0.05	0.02	2.72E-02		-0.26	0.07	1.26E-04	0.10
LDLR	ILMN_2053415	19	0.05	0.02	3.14E-02		0.27	0.07	1.34E-04	0.10
F11R	ILMN_2342033	1	-0.06	0.02	2.81E-03		-0.26	0.07	1.67E-04	0.11
MEF2C	ILMN_1742544	5	-0.06	0.02	3.49E-03		-0.27	0.07	1.86E-04	0.11
GAS6	ILMN_1779558	13	0.06	0.02	6.38E-03		0.26	0.07	2.11E-04	0.11
SHKBP1	ILMN_1765493	19	0.06	0.02	7.86E-03		0.27	0.07	1.69E-04	0.11
FER1L3	ILMN_2370976	10	0.06	0.02	8.68E-03		0.27	0.07	1.91E-04	0.11
CCNB1IP1	ILMN_2347349	14	-0.04	0.02	0.05		-0.27	0.07	2.19E-04	0.11
OLR1	ILMN_1723035	12	0.04	0.02	0.05		0.26	0.07	2.14E-04	0.11
MXD1	ILMN_2214678	2	0.04	0.02	0.05		0.28	0.07	1.60E-04	0.11
USP14	ILMN_1806804	18	-0.03	0.02	0.12		-0.26	0.07	2.07E-04	0.11
FAM160B2	ILMN_1802971	8	-0.01	0.02	0.75		0.26	0.07	2.06E-04	0.11
CDC42EP4	ILMN_1745223	17	0.00	0.02	0.93		0.29	0.08	2.15E-04	0.11
POLR1E	ILMN_2110167	9	-0.03	0.02	0.15		-0.26	0.07	2.48E-04	0.11
FGFRL1	ILMN_1795865	4	0.04	0.02	0.06		0.29	0.08	2.82E-04	0.12
SERBP1	ILMN_1773968	1	-0.03	0.02	0.13		-0.25	0.07	3.02E-04	0.12
DAB2	ILMN_1764228	5	0.02	0.02	0.37		0.25	0.07	3.03E-04	0.12
C19ORF39	ILMN_1707943	19	-0.02	0.02	0.26		-0.25	0.07	3.33E-04	0.13
RPN2	ILMN_1693421	20	-0.02	0.02	0.28		-0.25	0.07	3.43E-04	0.13
ALDH1A1	ILMN_2096372	9	-0.06	0.02	1.44E-03		-0.24	0.07	3.66E-04	0.13
CIAO1	ILMN_2048793	2	-0.05	0.02	1.33E-02		-0.26	0.07	4.01E-04	0.13
TPST2	ILMN_2329679	22	0.05	0.02	2.03E-02		0.26	0.07	3.94E-04	0.13
C6ORF225	ILMN_3248941	6	-0.03	0.02	0.12		-0.26	0.07	4.00E-04	0.13
COX17	ILMN_2187718	3	0.03	0.02	0.20		0.26	0.07	3.89E-04	0.13
SLC39A8	ILMN_1695316	4	0.09	0.02	3.67E-05	0.14	0.19	0.07	6.33E-03	
RBM38	ILMN_2404049	20	0.09	0.02	4.28E-05	0.14	0.12	0.07	0.09	
ALDOA	ILMN_1741148	16	0.09	0.02	4.84E-05	0.14	0.19	0.07	8.76E-03	
BLCAP	ILMN_1675612	20	-0.04	0.02	0.11		-0.26	0.07	4.44E-04	0.15
PAQR7	ILMN_1746618	1	-0.04	0.02	4.35E-02		-0.24	0.07	4.54E-04	0.15
FIGNL2	ILMN_3249343	12	-0.04	0.02	0.08		-0.24	0.07	4.86E-04	0.15
GGCT	ILMN_1745005	7	-0.05	0.02	2.96E-02		-0.25	0.07	5.06E-04	0.16
FDFT1	ILMN_2144088	8	0.07	0.02	7.31E-04		0.23	0.07	6.02E-04	0.16
CREBL2	ILMN_1660223	12	-0.04	0.02	4.87E-02		-0.24	0.07	6.22E-04	0.16
TCIRG1	ILMN_1711994	11	0.04	0.02	0.07		0.24	0.07	5.74E-04	0.16
CENTB2	ILMN_2088825	3	-0.04	0.02	0.07		-0.24	0.07	5.75E-04	0.16
RRAS	ILMN_1780825	19	0.03	0.02	0.24		0.26	0.08	6.12E-04	0.16
SNHG11	ILMN_1791884	20	0.02	0.02	0.39		0.24	0.07	5.78E-04	0.16
ENPP2	ILMN_2373791	8	0.01	0.02	0.53		0.24	0.07	5.71E-04	0.16

Gene			Carotid Plaque Score (n=1,181)				Coronary Artery Calcium (n=1,208)			
**Gene name**	**Illumina probe id**	**Chr**	**Beta**	**SE**	**P-value**	**FDR**	**Beta**	**SE**	**P-value**	**FDR**
AKR1B1	ILMN_1701731	7	-0.01	0.02	0.55		-0.25	0.07	6.22E-04	0.16
CCNB1IP1	ILMN_1752394	14	-0.01	0.02	0.68		-0.25	0.07	5.57E-04	0.16
SLC25A14	ILMN_2347044	X	-0.01	0.02	0.80		0.24	0.07	6.11E-04	0.16
CR1	ILMN_1767193	1	0.05	0.02	1.86E-02		0.24	0.07	6.97E-04	0.16
ELMO2	ILMN_2317730	20	0.05	0.02	2.53E-02		0.25	0.07	7.00E-04	0.16
ACAA1	ILMN_1738921	3	0.04	0.02	3.77E-02		0.24	0.07	7.17E-04	0.16
CHRAC1	ILMN_1722390	8	-0.03	0.02	0.13		-0.24	0.07	6.58E-04	0.16
RNF144B	ILMN_1752526	6	0.03	0.02	0.15		0.24	0.07	7.09E-04	0.16
PDIA5	ILMN_1695763	3	0.01	0.02	0.52		-0.24	0.07	6.62E-04	0.16
STX10	ILMN_1753712	19	0.01	0.02	0.76		0.24	0.07	6.83E-04	0.16
CDKN1A	ILMN_1784602	6	0.00	0.02	1.00		0.26	0.08	6.91E-04	0.16
RAB37	ILMN_2255579	17	-0.02	0.02	0.32		-0.25	0.07	7.37E-04	0.16
RAB37	ILMN_2243912	17	-0.04	0.02	4.56E-02		-0.25	0.07	7.72E-04	0.16
PRPF4	ILMN_1697440	9	-0.01	0.02	0.63		-0.23	0.07	7.78E-04	0.16
TPST2	ILMN_1695432	22	0.07	0.02	2.62E-03		0.24	0.07	8.73E-04	0.17
PIM1	ILMN_1815023	6	0.05	0.02	2.36E-02		0.25	0.07	8.11E-04	0.17
PFAS	ILMN_1755862	17	-0.03	0.02	0.10		-0.24	0.07	8.67E-04	0.17
ALKBH6	ILMN_1808792	19	-0.01	0.02	0.61		0.23	0.07	8.52E-04	0.17
ALOX15B	ILMN_2393149	17	-0.01	0.02	0.61		-0.24	0.07	8.39E-04	0.17
SET	ILMN_1742238	9	0.00	0.02	0.99		-0.24	0.07	8.52E-04	0.17
ATP2B1	ILMN_1813399	12	-0.09	0.02	6.91E-05	0.17	-0.09	0.07	0.24	
MYL6	ILMN_2326071	12	0.02	0.02	0.29		0.24	0.07	9.22E-04	0.18
ABCG1	ILMN_2329927	21	-0.06	0.02	2.39E-03		-0.23	0.07	9.35E-04	0.18
INPP1	ILMN_1667239	2	-0.08	0.02	8.82E-05	0.18	-0.16	0.07	1.96E-02	
MT1H	ILMN_2124802	16	0.08	0.02	9.82E-05	0.18	0.22	0.07	1.62E-03	
PGD	ILMN_1794165	1	0.09	0.02	1.09E-04	0.18	0.20	0.08	1.16E-02	
GALK2	ILMN_1723124	15	-0.09	0.02	1.37E-04	0.18	-0.15	0.08	4.85E-02	
PSMD1	ILMN_1702837	2	0.09	0.02	1.37E-04	0.18	0.06	0.07	0.40	
C10ORF58	ILMN_2118472	10	-0.08	0.02	1.41E-04	0.18	-0.03	0.07	0.64	
ABCG1	ILMN_1794782	21	-0.08	0.02	1.48E-04	0.18	-0.21	0.07	2.82E-03	
BCL2	ILMN_2246956	18	0.11	0.03	1.74E-04	0.18	0.16	0.10	0.09	
RPS19	ILMN_1784717	19	0.08	0.02	1.75E-04	0.18	0.13	0.07	0.06	
NICN1	ILMN_1807211	3	0.08	0.02	1.86E-04	0.18	0.18	0.07	1.14E-02	
MAPK13	ILMN_1749327	6	0.08	0.02	1.88E-04	0.18	0.20	0.07	5.43E-03	
PFDN2	ILMN_1678754	1	0.08	0.02	2.19E-04	0.18	0.16	0.07	2.28E-02	
SAMSN1	ILMN_1684887	21	0.08	0.02	2.27E-04	0.18	0.20	0.07	5.08E-03	
MID1IP1	ILMN_1668960	X	-0.08	0.02	2.30E-04	0.18	-0.14	0.07	0.06	
C1ORF24	ILMN_1667966	1	0.07	0.02	1.43E-03		0.23	0.07	9.70E-04	0.18
UBE2J1	ILMN_1713759	6	0.05	0.02	3.61E-02		0.25	0.07	9.84E-04	0.18
HIST3H2A	ILMN_1779648	1	0.04	0.02	4.78E-02		0.23	0.07	9.94E-04	0.18
ANAPC1	ILMN_1804812	2	-0.06	0.02	6.11E-03		-0.23	0.07	1.03E-03	0.18
CRTAP	ILMN_1720484	3	-0.04	0.02	0.06		-0.24	0.07	1.02E-03	0.18
RASSF2	ILMN_2352303	20	0.00	0.02	0.92		-0.24	0.07	1.03E-03	0.18
PDCD4	ILMN_1768004	10	-0.05	0.02	2.34E-02		-0.23	0.07	1.06E-03	0.18
FDFT1	ILMN_1741096	8	0.07	0.02	2.44E-04	0.18	0.04	0.07	0.55	
LYZ	ILMN_2162972	12	0.06	0.02	1.36E-02		0.25	0.08	1.25E-03	0.19
PIP5K1B	ILMN_1736654	9	-0.04	0.02	0.05		-0.23	0.07	1.18E-03	0.19
RAB27A	ILMN_2329773	15	-0.03	0.02	0.12		-0.24	0.08	1.23E-03	0.19
NCF1C	ILMN_2112988	7	0.03	0.02	0.13		0.23	0.07	1.24E-03	0.19
TMEM19	ILMN_3251207	12	-0.03	0.02	0.14		-0.24	0.07	1.15E-03	0.19
DLEU7	ILMN_3307752	13	-0.02	0.02	0.30		-0.24	0.08	1.26E-03	0.19
ZBTB17	ILMN_1711048	1	0.02	0.02	0.45		0.23	0.07	1.24E-03	0.19
EIF4EBP3	ILMN_2197225	5	-0.01	0.02	0.66		-0.23	0.07	1.27E-03	0.19
METAP2	ILMN_1670420	12	0.00	0.02	0.98		-0.23	0.07	1.26E-03	0.19
ARAP2	ILMN_3244434	4	0.05	0.02	2.85E-02		0.23	0.07	1.31E-03	0.19
NR3C1	ILMN_2389347	5	-0.04	0.02	4.84E-02		-0.23	0.07	1.31E-03	0.19
PTPRO	ILMN_1720113	12	0.03	0.02	0.12		0.24	0.07	1.30E-03	0.19

Gene			Carotid Plaque Score (n=1,181)				Coronary Artery Calcium (n=1,208)			
**Gene name**	**Illumina probe id**	**Chr**	**Beta**	**SE**	**P-value**	**FDR**	**Beta**	**SE**	**P-value**	**FDR**
C10ORF32	ILMN_1772706	10	-0.03	0.02	0.10		-0.23	0.07	1.33E-03	0.19
STX12	ILMN_1773901	1	-0.04	0.02	0.06		-0.23	0.07	1.35E-03	0.19
ALPK1	ILMN_2078697	4	0.06	0.02	5.23E-03		0.23	0.07	1.41E-03	0.20
FAM63A	ILMN_1689552	1	-0.05	0.02	4.11E-02		-0.25	0.08	1.40E-03	0.20
ZNF770	ILMN_1734254	15	0.00	0.02	0.88		-0.23	0.07	1.41E-03	0.20
CALML4	ILMN_1717757	15	0.04	0.02	0.08		0.22	0.07	1.44E-03	0.20
SLC26A6	ILMN_1785252	3	0.00	0.02	0.83		0.23	0.07	1.45E-03	0.20
LAIR1	ILMN_1768598	19	0.06	0.02	7.62E-03		0.22	0.07	1.49E-03	0.20
ATXN10	ILMN_2169676	22	-0.05	0.02	1.79E-02		-0.22	0.07	1.47E-03	0.20
FBL	ILMN_1719205	19	0.03	0.02	0.15		-0.24	0.08	1.50E-03	0.20
CRY1	ILMN_2202637	12	-0.04	0.02	0.05		-0.22	0.07	1.52E-03	0.20

Sorted by most significant association (FDR) with carotid plaque burden or CAC; FDR ≥0.20 are left blank.

Supplementary Table 2. Race/ethnicity and sex-stratified analysis of genes with mRNA expression associated with Atherosclerosis

Carotid Plaque Score associated gene expression (FDR≤0.05):

	African American (N=260)			Caucasian (N=532)			Hispanic (N=372)			Women (N=603)			Men (N=561)		
**Gene**	**Beta**	**SE**	**P-val**	**Beta**	**SE**	**P-val**	**Beta**	**SE**	**P-val**	**Beta**	**SE**	**P-val**	**Beta**	**SE**	**P-val**
ARID5B	0.13	0.06	0.019	0.15	0.03	2.3E-06	0.06	0.04	0.17	0.08	0.03	0.014	0.14	0.03	1.0E-05
PDLIM7	0.05	0.05	0.27	0.09	0.03	2.6E-03	0.08	0.04	0.034	0.10	0.03	8.6E-04	0.05	0.03	0.13

Coronary artery calcium associated gene expression (FDR≤0.05):

	African American (N=259)			Caucasian (N=544)			Hispanic (N=387)			Women (N=613)			Men (N=577)		
**Gene**	**Beta**	**SE**	**P-val**	**Beta**	**SE**	**P-val**	**Beta**	**SE**	**P-val**	**Beta**	**SE**	**P-val**	**Beta**	**SE**	**P-val**
CACNA2D3	-0.64	0.16	7.4E-05	-0.25	0.12	0.029	-0.26	0.14	0.078	-0.12	0.11	0.28	-0.56	0.12	1.6E-06
ARID5B	0.36	0.19	0.057	0.14	0.10	0.16	0.20	0.14	0.15	0.06	0.11	0.56	0.33	0.11	1.9E-03
PDLIM7	0.07	0.15	0.63	0.21	0.10	0.042	0.27	0.13	0.033	0.11	0.10	0.28	0.26	0.10	0.010
TSPYL1	-0.28	0.14	0.054	-0.28	0.09	2.4E-03	-0.19	0.14	0.15	-0.28	0.08	8.9E-04	-0.29	0.11	9.5E-03
RSU1	-0.32	0.16	0.044	-0.19	0.10	0.056	-0.24	0.12	0.047	-0.27	0.09	3.6E-03	-0.19	0.10	0.067
LILRB4	0.39	0.14	6.1E-03	0.23	0.09	0.014	0.29	0.14	0.032	0.23	0.09	0.011	0.33	0.10	9.7E-04
P2RX4	0.20	0.16	0.22	0.36	0.10	2.3E-04	0.10	0.12	0.37	0.22	0.09	0.016	0.26	0.10	9.4E-03
CANX	-0.19	0.16	0.22	-0.32	0.10	2.1E-03	-0.21	0.13	0.12	-0.31	0.09	8.6E-04	-0.23	0.11	0.034
FAM168B	-0.27	0.16	0.090	-0.24	0.10	0.013	-0.26	0.12	0.038	-0.24	0.09	7.4E-03	-0.29	0.11	5.6E-03
ADM	0.29	0.14	0.045	0.26	0.11	0.016	0.27	0.14	0.049	0.17	0.10	0.084	0.39	0.11	2.3E-04
IARS	-0.30	0.17	0.078	-0.21	0.10	0.032	-0.23	0.12	0.061	-0.18	0.09	0.055	-0.30	0.10	3.6E-03
FAM10A4	-0.36	0.15	0.016	-0.18	0.10	0.065	-0.19	0.13	0.16	-0.30	0.09	1.1E-03	-0.14	0.10	0.17
EIF5A2	0.32	0.14	0.025	0.30	0.10	2.5E-03	0.04	0.13	0.76	0.24	0.09	6.8E-03	0.18	0.11	0.098

Supplementary Table 3. Gene co-expression networks associated with subclinical atherosclerosis

Module			†Carotid Plaque Score			†Coronary Artery Calcium		
**Module name**	Genes	***Enriched Bio-functions**	**Beta**	**SE**	**P-value**	**Beta**	**SE**	**P-value**
Module 35	LDLR, SC5DL, FADS1, CYP51A1, FDFT1, SCD, MYLIP, SC4MOL, HMGCS1, SQLE, ABCG1, ABCA1	Steroid metabolism (FDR = 4.23E-11; LDLR, CYP51A1, SQLE, HMGCS1, ABCA1, ABCG1, SC5DL, FDFT1, SC4MOL)	**0.08**	**0.02**	**2.31E-04**	**0.25**	**0.07**	**3.34E-04**
Module 39	PIM1, GAS6, MTHFD2, FCGR1B, FCGR1A, FCGR1C, CASP5	Phagosome formation (FDR =1.32E-02; FCGR1C, FCGR1A, FCGR1B)	0.06	0.02	4.62E-03	**0.24**	**0.07**	**5.26E-04**
Module 23	OSBPL8, CCDC109A, CX3CR1, CBARA1, RRAGD, RAPH1, FAM129A, UBE2J1, HDAC9, EPS8, ITGB5, C1ORF24, LPCAT3, CDK6, ELMO2, TPST2, SORT1, KCNMB4, PLA2G15, P2RX4, SNTB1, SLITRK4, PDGFC, MYD88, MPHOSPH6, TCF4	Migration of cells (FDR = 3.96E-02; ITGB5, EPS8, CX3CR1, MYD88, CDK6, OSBPL8, ELMO2, TCF4, SORT1)	0.05	0.02	0.02	**0.22**	**0.07**	**1.63E-03**

*Enrichment from IPA Ingenuity, module genes compared to a background of all genes with detectable expression; †Module eigengene associations FDR_BH ≤ 0.05 shown in bold.

Supplementary Table 4. Monocyte CpGs with Methylation Associated with Atherosclerosis (FDR ≤ 0.10)

CpG methylation characteristics								Methylation ~ Carotid Plaque (N=1,176)				Methylation ~ CAC (N=1,203)			
**CpG ID**	**Chr**	**Location**	**CpG island**	***TFBS**	**†DMR**	**‡DNase HS**	**‡Predicted state in B cell line (GM12878)**	**Beta**	**SE**	**P-value**	**FDR**	**Beta**	**SE**	**P-value**	**FDR**
**cg06126421**	6	Intergenic		1		1	13 Heterochrom/lo	-0.14	0.02	2.00E-10	8.91E-05	-0.19	0.07	8.33E-03	
**cg05575921**	5	AHRR (Body)	Shore	1			4 Strong Enhancer	-0.13	0.02	4.05E-10	9.01E-05	-0.25	0.07	3.40E-04	
**cg21566642**	2	Intergenic	Island		DMR		12 Repressed	-0.12	0.02	3.99E-09	5.92E-04	-0.27	0.07	1.44E-04	
**cg05951221**	2	Intergenic	Island			1	12 Repressed	-0.12	0.02	2.15E-08	2.40E-03	-0.26	0.07	1.34E-04	
**cg06644428**	2	Intergenic	Island		DMR		12 Repressed	-0.11	0.02	6.44E-08	5.73E-03	-0.26	0.07	2.04E-04	
**cg05119988**	4	SC4MOL (5'UTR)	Shelf	1			2 Weak Promoter	-0.08	0.02	1.63E-04		-0.36	0.07	9.83E-08	0.03
**cg19572487**	17	RARA (5'UTR)	Shore	1		1	1 Active Promoter	-0.11	0.02	9.89E-08	7.33E-03	-0.20	0.07	4.89E-03	
**cg23661483**	19	ILVBL (Body)	Shelf				11 Weak Txn	0.11	0.02	6.09E-07	0.02	0.38	0.07	1.28E-07	0.03
**cg14753356**	6	Intergenic		1		1	13 Heterochrom/lo	-0.11	0.02	1.43E-07	9.09E-03	-0.15	0.07	0.04	
**cg09935388**	1	GFI1 (Body)	Island		CDMR	1	11 Weak Txn	-0.11	0.02	1.77E-07	9.84E-03	-0.17	0.07	0.01	
**cg05546038**	16	NOL3 (TSS1500)	Shore	1			11 Weak Txn	0.11	0.02	2.05E-07	0.01	0.16	0.07	0.03	
**cg10549018**	10	TLL2 (Body)					7 Weak Enhancer	0.06	0.02	5.08E-03		0.36	0.07	2.28E-07	0.03
**cg21161138**	5	AHRR (Body)		1			5 Strong Enhancer	-0.11	0.02	2.78E-07	0.01	-0.21	0.07	2.86E-03	
**cg15342087**	6	Intergenic		1		1	13 Heterochrom/lo	-0.11	0.02	2.79E-07	0.01	-0.06	0.07	0.36	
**cg03295554**	11	ETS1 (Body)	Shelf	1		1	5 Strong Enhancer	-0.10	0.02	3.10E-07	0.01	-0.20	0.07	3.22E-03	
**cg14026106**	10	MARCH8 (Body)		1		1	13 Heterochrom/lo	-0.06	0.02	3.19E-03		-0.36	0.07	3.26E-07	0.03
**cg24859433**	6	Intergenic		1		1	13 Heterochrom/lo	-0.11	0.02	3.57E-07	0.01	-0.10	0.07	0.18	
**cg00889709**	8	FAM110B (5'UTR)				1	13 Heterochrom/lo	0.03	0.02	0.10		0.36	0.07	3.83E-07	0.03
**cg25953130**	10	ARID5B (Body)		1		1	5 Strong Enhancer	-0.11	0.02	4.31E-07	0.01	-0.28	0.07	6.80E-05	
**cg00021275**	2	Intergenic	Shelf			1	9 Txn Transition	0.11	0.02	4.39E-07	0.01	0.23	0.08	2.05E-03	
**cg24247537**	11	PTDSS2 (Body)	Shore				4 Strong Enhancer	0.06	0.02	0.01		0.37	0.07	4.84E-07	0.03
**cg18857467**	1	HDAC1 (Body)		1		1	4 Strong Enhancer	-0.07	0.02	2.01E-03		-0.35	0.07	4.89E-07	0.03
**cg16107001**	4	C4orf48 (TSS1500)	Island				2 Weak Promoter	0.10	0.02	5.00E-07	0.01	0.10	0.07	0.13	
**cg21271420**	6	Intergenic					13 Heterochrom/lo	-0.11	0.02	6.49E-07	0.02	-0.05	0.07	0.50	
**cg08867471**	5	Intergenic		1		1	13 Heterochrom/lo	-0.12	0.02	9.09E-07	0.02	-0.08	0.08	0.31	
**cg03636183**	19	F2RL3 (Body)	Shore	1		1	6 Weak Enhancer	-0.10	0.02	9.17E-07	0.02	-0.16	0.07	0.02	
**cg01940273**	2	Intergenic	Island	1			12 Repressed	-0.10	0.02	9.21E-07	0.02	-0.22	0.07	1.56E-03	
**cg16663980**	1	MNDA (5'UTR)					11 Weak Txn	0.07	0.02	1.33E-03		0.34	0.07	1.04E-06	0.06
**cg04761231**	9	RPL35 (Body)	Island	1		1	1 Active Promoter	-0.11	0.02	1.23E-06	0.02	-0.27	0.08	3.09E-04	
**cg19266329**	1	Intergenic		1		1	4 Strong Enhancer	-0.10	0.02	1.34E-06	0.03	-0.18	0.07	0.01	
**cg15501219**	3	Intergenic	Shelf				13 Heterochrom/lo	0.13	0.03	1.67E-06	0.03	0.42	0.09	5.15E-06	
**cg24574691**	11	Intergenic					13 Heterochrom/lo	0.10	0.02	1.83E-06	0.03	0.17	0.07	0.01	
**cg00834988**	7	AUTS2 (Body)	Shore				13 Heterochrom/lo	0.10	0.02	1.84E-06	0.03	0.29	0.07	4.65E-05	
**cg02160023**	16	CLCN7 (3'UTR)	Shore				13 Heterochrom/lo	0.02	0.02	0.42		0.33	0.07	2.01E-06	0.10
**cg24420089**	11	PTDSS2 (Body)	Shore				4 Strong Enhancer	0.05	0.02	0.03		0.34	0.07	2.27E-06	0.10

		CpG methylation characteristics						Methylation ~ Carotid Plaque (N=1,176)				Methylation ~ CAC (N=1,203)			
**CpG ID**	**Chr**	**Location**	**CpG island**	***TFBS**	**†DMR**	**‡DNase HS**	**‡Predicted state in B cell line (GM12878)**	**Beta**	**SE**	**P-value**	**FDR**	**Beta**	**SE**	**P-value**	**FDR**
**cg13200854**	4	MMRN1 (Body)				1	13 Heterochrom/lo	-0.10	0.02	2.27E-06	0.04	-0.24	0.07	7.39E-04	
**cg07033253**	7	SRRM3 (Body)	Shore				13 Heterochrom/lo	-0.09	0.02	1.05E-05	0.09	-0.33	0.07	2.44E-06	0.10
**cg05246522**	17	KSR1 (TSS200)		1		1	4 Strong Enhancer	-0.07	0.02	6.58E-04		-0.35	0.07	2.70E-06	0.10
**cg09076584**	17	FLJ25006 (TSS200)				1	10 Txn Elongation	0.08	0.02	1.24E-04		0.33	0.07	2.74E-06	0.10
**cg08958747**	16	RAB26 (Body)	Shelf	1		1	4 Strong Enhancer	-0.10	0.02	2.83E-06	0.04	-0.17	0.07	0.02	
**cg23848152**	1	NOTCH2NL (5'UTR)	Shore				3 Poised Promoter	-0.10	0.02	2.93E-06	0.04	-0.15	0.07	0.03	
**cg12798040**	14	XRCC3 (Body)		1		1	10 Txn Elongation	0.07	0.02	7.47E-04		0.32	0.07	3.29E-06	0.10
**cg13913475**	X	Intergenic	Shore	1		1	8 Insulator	-0.05	0.02	0.01		-0.32	0.07	3.31E-06	0.10
**cg13699363**	3	EDEM1 (TSS1500)	Island	1		1	1 Active Promoter	-0.04	0.02	0.04		-0.32	0.07	3.62E-06	0.10
**cg24736734**	5	Intergenic		1		1	7 Weak Enhancer	-0.10	0.02	3.66E-06	0.05	-0.21	0.07	4.02E-03	
**cg01763719**	2	Intergenic					13 Heterochrom/lo	0.11	0.02	3.70E-06	0.05	0.22	0.08	9.20E-03	
**cg03355101**	19	Intergenic		1	DMR		2 Weak Promoter	-0.09	0.02	3.99E-06	0.06	0.04	0.07	0.59	
**cg10512376**	4	EMCN (TSS1500)					13 Heterochrom/lo	-0.10	0.02	5.71E-06	0.08	-0.24	0.07	9.98E-04	
**cg22773522**	13	DAOA (TSS1500)					13 Heterochrom/lo	-0.09	0.02	6.19E-06	0.08	-0.08	0.07	0.25	
**cg18446336**	7	GNA12 (Body)		1		1	11 Weak Txn	-0.09	0.02	6.45E-06	0.08	-0.07	0.07	0.31	
**cg10508317**	17	SOCS3 (Body)	Island	1	DMR	1	3 Poised Promoter	-0.10	0.02	6.98E-06	0.09	-0.20	0.07	7.12E-03	
**cg00294684**	11	OR52B6 (1stExon)					13 Heterochrom/lo	-0.09	0.02	7.48E-06	0.09	-0.16	0.07	0.02	
**cg02475408**	7	Intergenic	Shore				13 Heterochrom/lo	-0.09	0.02	7.74E-06	0.09	-0.18	0.07	7.67E-03	
**cg05888181**	8	KBTBD11 (3'UTR)	Shore			1	13 Heterochrom/lo	0.11	0.02	7.90E-06	0.09	0.14	0.08	0.09	
**cg20507228**	15	MAN2A2 (Body)					10 Txn Elongation	0.09	0.02	7.91E-06	0.09	0.29	0.07	3.84E-05	
**cg03738331**	4	HTT (TSS200)	Island	1		1	1 Active Promoter	-0.09	0.02	8.24E-06	0.09	-0.12	0.07	0.07	
**cg01692968**	9	Intergenic	Shore	1		1	6 Weak Enhancer	-0.10	0.02	8.46E-06	0.09	-0.11	0.08	0.15	
**cg23840027**	1	EPB41 (5'UTR)					11 Weak Txn	0.09	0.02	8.49E-06	0.09	0.15	0.07	0.04	
**cg09768249**	X	DDX3X (TSS200)	Island	1		1	1 Active Promoter	0.09	0.02	8.50E-06	0.09	0.003	0.069	0.97	
**cg09656152**	5	Intergenic					13 Heterochrom/lo	-0.09	0.02	8.69E-06	0.09	-0.08	0.07	0.23	
**cg16661609**	19	LILRB4 (TSS1500)					5 Strong Enhancer	-0.09	0.02	9.47E-06	0.09	-0.13	0.07	0.08	
**cg03072035**	10	Intergenic	Shelf			1	13 Heterochrom/lo	0.10	0.02	9.55E-06	0.09	0.17	0.08	0.02	
**cg01110839**	11	SLC39A13 (3'UTR)		1		1	11 Weak Txn	0.10	0.02	1.04E-05	0.09	0.23	0.07	1.63E-03	
**cg08170227**	14	ACTN1 (Body)		1		1	11 Weak Txn	-0.09	0.02	1.05E-05	0.09	-0.21	0.07	2.14E-03	
**cg00913954**	1	STK40 (TSS1500)	Shore	1	RDMR	1	4 Strong Enhancer	0.11	0.02	1.05E-05	0.09	0.14	0.08	0.08	
**cg19979108**	5	SH3PXD2B (Body)		1			12 Repressed	-0.10	0.02	1.11E-05	0.09	-0.18	0.07	0.02	
**cg05249393**	19	LDLR (TSS200)	Shore	1		1	4 Strong Enhancer	-0.09	0.02	1.12E-05	0.09	-0.16	0.07	0.02	
**cg15344028**	2	ICOS (5'UTR)					13 Heterochrom/lo	-0.09	0.02	1.13E-05	0.09	-0.13	0.07	0.06	
**cg12912426**	12	ATF7IP (5'UTR)					5 Strong Enhancer	-0.09	0.02	1.15E-05	0.09	-0.12	0.07	0.08	
**cg05850338**	17	Intergenic	Shelf				13 Heterochrom/lo	0.09	0.02	1.16E-05	0.09	0.11	0.07	0.13	
**cg18561976**	2	ICOS (5'UTR)				1	13 Heterochrom/lo	-0.09	0.02	1.18E-05	0.09	-0.17	0.07	0.01	

CpG methylation characteristics								Methylation ~ Carotid Plaque (N=1,176)				Methylation ~ CAC (N=1,203)			
**CpG ID**	**Chr**	**Location**	**CpG island**	***TFBS**	**†DMR**	**‡DNase HS**	**‡Predicted state in B cell line (GM12878)**	**Beta**	**SE**	**P-value**	**FDR**	**Beta**	**SE**	**P-value**	**FDR**
**cg03059174**	20	SFRS6 (TSS1500)	Shore	1		1	1 Active Promoter	-0.09	0.02	1.18E-05	0.09	-0.10	0.07	0.14	
**cg12547807**	1	Intergenic		1		1	4 Strong Enhancer	-0.09	0.02	1.19E-05	0.09	-0.20	0.07	3.79E-03	
**cg17038626**	12	CACNA1C (Body)					12 Repressed	-0.09	0.02	1.20E-05	0.09	-0.06	0.07	0.36	
**cg26712743**	10	PYROXD2 (Body)		1		1	4 Strong Enhancer	-0.10	0.02	1.22E-05	0.09	-0.24	0.07	1.25E-03	
**cg18168448**	1	LRRC52 (1stExon)					12 Repressed	-0.09	0.02	1.27E-05	0.09	-0.17	0.07	0.01	
**cg09646173**	5	PDE6A (Body)		1			13 Heterochrom/lo	-0.10	0.02	1.32E-05	0.09	-0.24	0.07	1.39E-03	
**cg16006965**	3	GCET2 (TSS1500)		1		1	4 Strong Enhancer	-0.09	0.02	1.34E-05	0.09	-0.07	0.07	0.29	
**cg08882503**	1	NPHP4 (Body)				1	11 Weak Txn	-0.09	0.02	1.38E-05	0.09	-0.12	0.07	0.08	
**cg16016063**	1	Intergenic					13 Heterochrom/lo	0.09	0.02	1.40E-05	0.09	0.15	0.07	0.03	
**cg03928367**	20	FER1L4 (Body)	Shore		CDMR	1	12 Repressed	0.09	0.02	1.45E-05	0.10	0.11	0.07	0.11	
**cg18050903**	4	RASGEF1B (Body)		1		1	13 Heterochrom/lo	-0.09	0.02	1.56E-05	0.10	-0.18	0.07	0.01	

Sorted by most significant association (p-value) with carotid plaque or CAC; FDR > 0.10 are left blank

*TFBS in any cell type from UCSC browser; † DMR: diff methylated region from Illumina (RDMR: reprogramming-specific, CDMR: cancer-specific);

‡DNase HS in any cell type and predicted state from ENCODE.

Supplementary Table 5. Race/ethnicity and sex-stratified analysis of CpGs with methylation associated with Atherosclerosis

Carotid Plaque Score associated CpG methylation (FDR≤0.05):

CpG Methylation		African American (N=259)			Caucasian (N=530)			Hispanic (N=370)			Women (N=601)			Men (N=558)		
CpG ID	Location	Beta	SE	P-val	Beta	SE	P-val	Beta	SE	P-val	Beta	SE	P-val	Beta	SE	P-val
cg16107001	C4orf48 (TSS1500)	0.10	0.05	0.026	0.14	0.03	2.3E-06	0.05	0.03	0.15	0.06	0.03	0.020	0.14	0.03	9.7E-07
cg21271420	Intergenic	-0.07	0.04	0.10	-0.12	0.03	9.4E-05	-0.09	0.04	0.029	-0.09	0.03	2.2E-03	-0.12	0.03	1.6E-04
cg23848152	NOTCH2NL (5'UTR)	-0.13	0.05	5.4E-03	-0.09	0.03	1.2E-03	-0.07	0.03	0.061	-0.05	0.03	0.065	-0.15	0.03	1.6E-06
cg08867471	Intergenic	-0.04	0.05	0.37	-0.17	0.04	2.2E-06	-0.07	0.04	0.11	-0.13	0.03	7.6E-05	-0.10	0.03	5.3E-03
cg05575921	AHRR (Body)	-0.05	0.06	0.38	-0.17	0.04	1.0E-05	-0.09	0.05	0.077	-0.05	0.04	0.20	-0.16	0.04	7.1E-06
cg25953130	ARID5B (Body)	-0.08	0.05	0.073	-0.13	0.03	6.3E-05	-0.04	0.04	0.31	-0.10	0.03	7.3E-04	-0.09	0.03	5.9E-03
cg06126421	Intergenic	-0.09	0.06	0.10	-0.14	0.03	4.4E-05	-0.05	0.04	0.19	-0.06	0.03	0.080	-0.16	0.03	5.8E-06
cg05546038	NOL3 (TSS1500)	0.10	0.04	0.022	0.11	0.03	1.2E-03	0.09	0.04	0.014	0.08	0.03	7.7E-03	0.11	0.03	4.0E-04
cg24574691	Intergenic	0.07	0.03	0.035	0.09	0.05	0.072	0.09	0.03	9.8E-04	0.08	0.02	1.8E-04	0.08	0.04	0.036
cg09935388	GFI1 (Body)	-0.11	0.05	0.035	-0.11	0.03	1.6E-04	-0.03	0.04	0.50	-0.08	0.03	9.5E-03	-0.10	0.03	1.6E-03
cg01763719	Intergenic	0.13	0.05	0.019	0.10	0.04	7.9E-03	0.10	0.04	0.017	0.15	0.03	2.1E-05	0.07	0.03	0.038
cg00021275	Intergenic	0.12	0.06	0.032	0.09	0.03	7.7E-03	0.10	0.04	8.7E-03	0.11	0.03	2.4E-04	0.07	0.03	0.025
cg23661483	ILVBL (Body)	0.07	0.05	0.13	0.06	0.03	0.058	0.12	0.04	7.7E-04	0.11	0.03	1.7E-04	0.07	0.03	0.022
cg08958747	RAB26 (Body)	-0.10	0.05	0.049	-0.09	0.03	8.0E-03	-0.08	0.04	0.039	-0.08	0.03	9.9E-03	-0.10	0.03	1.5E-03
cg13200854	MMRN1 (Body)	-0.08	0.05	0.11	-0.11	0.03	1.4E-04	-0.06	0.04	0.083	-0.04	0.03	0.14	-0.13	0.03	1.2E-05
cg24736734	Intergenic	-0.14	0.05	3.1E-03	-0.07	0.03	0.023	-0.11	0.04	7.6E-03	-0.11	0.03	9.3E-05	-0.07	0.03	0.024
cg19572487	RARA (5'UTR)	-0.04	0.05	0.46	-0.12	0.03	1.9E-04	-0.06	0.04	0.14	-0.05	0.03	0.11	-0.12	0.03	7.6E-05
cg03295554	ETS1 (Body)	-0.08	0.04	0.061	-0.09	0.03	2.6E-03	-0.08	0.04	0.028	-0.10	0.03	3.1E-04	-0.06	0.03	0.045
cg19266329	Intergenic	-0.14	0.05	3.2E-03	-0.10	0.03	1.4E-03	-0.02	0.04	0.68	-0.07	0.03	0.015	-0.10	0.03	1.3E-03
cg04761231	RPL35 (Body)	-0.06	0.05	0.22	-0.12	0.03	2.2E-04	-0.05	0.04	0.27	-0.07	0.03	0.016	-0.11	0.03	1.4E-03
cg21566642	Intergenic	-0.07	0.06	0.20	-0.11	0.03	2.1E-03	-0.06	0.04	0.16	-0.06	0.03	0.067	-0.12	0.03	5.0E-04
cg15501219	Intergenic	0.13	0.06	0.037	0.06	0.04	0.13	0.13	0.04	4.0E-03	0.11	0.04	7.0E-03	0.10	0.04	4.6E-03
cg15342087	Intergenic	-0.09	0.04	0.039	-0.09	0.03	9.9E-03	-0.03	0.04	0.42	-0.07	0.03	0.035	-0.09	0.03	3.7E-03
cg00834988	AUTS2 (Body)	0.05	0.04	0.24	0.06	0.03	0.043	0.10	0.03	2.8E-03	0.08	0.03	6.4E-03	0.07	0.03	0.024
cg24859433	Intergenic	-0.08	0.05	0.077	-0.09	0.03	8.0E-03	-0.02	0.04	0.62	-0.07	0.03	0.029	-0.09	0.03	7.5E-03
cg14753356	Intergenic	-0.06	0.05	0.28	-0.11	0.03	1.5E-03	-0.04	0.04	0.32	-0.03	0.03	0.42	-0.13	0.03	5.9E-05
cg21161138	AHRR (Body)	-0.13	0.05	8.8E-03	-0.09	0.03	9.9E-03	-0.03	0.04	0.54	-0.05	0.03	0.13	-0.11	0.03	1.4E-03
cg05951221	Intergenic	-0.04	0.06	0.48	-0.09	0.03	5.9E-03	-0.05	0.04	0.23	-0.06	0.03	0.077	-0.10	0.03	5.9E-03
cg06644428	Intergenic	-0.07	0.05	0.15	-0.06	0.03	0.063	-0.07	0.04	0.086	-0.04	0.03	0.25	-0.10	0.03	1.4E-03
cg03636183	F2RL3 (Body)	0.00	0.04	0.95	-0.13	0.04	4.1E-04	-0.03	0.04	0.40	-0.03	0.03	0.35	-0.10	0.03	1.2E-03
cg01940273	Intergenic	-0.08	0.05	0.16	-0.09	0.03	9.5E-03	-0.02	0.04	0.59	-0.04	0.03	0.27	-0.10	0.03	2.8E-03

Coronary artery calcium associated CpG methylation (FDR≤0.05):																	
		African American (N=258)			Caucasian (N=542)			Hispanic (N=385)			Women (N=611)			Men (N=574)		
CpG ID	Location	Beta	SE	P-val	Beta	SE	P-val	Beta	SE	P-val	Beta	SE	P-val	Beta	SE	P-val
cg18857467	HDAC1 (Body)	-0.25	0.17	0.15	-0.31	0.09	4.6E-04	-0.18	0.13	0.16	-0.26	0.09	4.0E-03	-0.23	0.10	0.022
cg05119988	SC4MOL (5'UTR)	-0.40	0.14	5.9E-03	-0.23	0.10	0.016	-0.07	0.13	0.56	-0.17	0.09	0.054	-0.21	0.10	0.037
cg00889709	FAM110B (5'UTR)	0.19	0.13	0.15	0.21	0.11	0.062	0.35	0.12	3.5E-03	0.17	0.10	0.080	0.30	0.10	2.8E-03
cg14026106	42071 (Body)	-0.22	0.12	0.076	-0.25	0.11	0.021	-0.40	0.14	4.4E-03	-0.20	0.09	0.035	-0.30	0.10	4.1E-03
cg10549018	TLL2 (Body)	0.20	0.15	0.20	0.26	0.10	0.011	0.42	0.12	3.7E-04	0.35	0.09	1.5E-04	0.26	0.10	0.011
cg24247537	PTDSS2 (Body)	0.47	0.14	1.3E-03	0.22	0.11	0.039	0.34	0.13	8.3E-03	0.44	0.10	6.7E-06	0.18	0.11	0.084
cg23661483	ILVBL (Body)	0.36	0.16	0.029	0.23	0.10	0.021	0.36	0.13	4.9E-03	0.33	0.10	5.5E-04	0.27	0.10	8.7E-03

Sorted by most significant association (p-value) with carotid plaque burden or CAC

Supplementary Table 6. DNA methylation associated with atherosclerosis and cis-gene expression

CpG methylation characteristics						CpG methylation ~ Cis-gene expression (N=1,264)				mRNA expression ~ Carotid Plaque Score (N=1,181)				mRNA expression ~ CAC (N=1,208)			
CpG Illumina ID	chromosome	Location	CD14+ in silico predicted state*	CD14+ DNase HS †	~ Carotid plaque (CP) or CAC FDR<0.05	Gene	corr‡	P-value	FDR	Beta	SE	**P-value**	FDR	Beta	SE	**P-value**	FDR
cg25953130	10	ARID5B (Body)	5	1	CP	***ARID5B***	-0.21	1.3E-08	5.7E-06	0.12	0.02	**6.3E-08**	1.1E-03	0.32	0.08	**2.5E-05**	0.034
cg05119988	4	SC4MOL (5'UTR)	2		CAC	***SC4MOL***	-0.17	1.2E-10	7.3E-08	0.07	0.02	**1.4E-03**	0.31	0.17	0.07	**0.014**	0.31
cg05546038	16	NOL3 (TSS1500)	6	1	CP	*DPEP2*	-0.21	1.5E-11	1.0E-08	-0.06	0.02	**7.6E-03**	0.41	-0.01	0.07	**0.91**	0.82
cg05546038	16	NOL3 (TSS1500)	6	1	CP	*ZDHHC1*	-0.19	1.5E-10	9.1E-08	-0.05	0.02	**0.030**	0.50	-0.03	0.07	**0.67**	0.77
cg05546038	16	NOL3 (TSS1500)	6	1	CP	*ATP6V0D1*	0.08	2.4E-06	6.5E-04	0.03	0.02	**0.12**	0.64	0.04	0.07	**0.63**	0.76
cg05546038	16	NOL3 (TSS1500)	6	1	CP	*NOL3*	-0.18	2.2E-09	1.1E-06	-0.01	0.02	**0.53**	0.86	0.05	0.07	**0.49**	0.71
cg05546038	16	NOL3 (TSS1500)	6	1	CP	*DYNC1LI2*	-0.12	1.6E-06	4.4E-04	-0.01	0.02	**0.75**	0.90	0.01	0.07	**0.91**	0.82
cg24736734	5		2		CP	*PAM*	-0.13	1.2E-08	5.1E-06	0.04	0.02	**0.066**	0.56	0.09	0.07	**0.19**	0.57
cg00889709	8	FAM110B (5'UTR)	3	1	CAC	*FAM110B*	-0.14	3.8E-08	1.5E-05	-0.01	0.02	**0.66**	0.89	-0.02	0.07	**0.81**	0.80

Table includes CpGs with significant (FDR<0.05) associations between CD14+ CpG methylation and atherosclerosis traits carotid plaque or coronary artery calcium (CAC), and *cis* (± 1 MB) gene expression profiles (in CD14+ samples from MESA, FDR<0.001)

*States predicted using ChromHMM with CD14+ histone data from BLUEPRINT; state annotations are 1: transcribed, 2: heterochromatin/low abundance, 3: weak/poised enhancer, 4: strong enhancer, 5: active promoter, 6: weak promoter

† DNase hypersensitive sites in a CD14+ sample from BLUEPRINT

‡correlation between methylation and cis (± 1MB) mRNA expression profiles

Supplementary Table 7. Atherosclerosis-associated gene expression in monocytes associations with CVD risk factors*

Gene	~ Carotid plaque (CP) or CAC FDR<0.05	Beta direction (+/-) or P-val	Age	Sex (male)	Race (Black)	Race (Hispanic)	Current smoker	BMI	HDL cholesterol	Hypertension	Diabetes
CACNA2D3	CAC	+ -	-		-	-		-	+	-	-
		P-val	1.81E-10		7.75E-25	3.32E-15		8.47E-12	5.33E-04	4.96E-05	5.55E-05
ARID5B	CP, CAC	+ -	+	+	+	+	+	+	-	+	+
		P-val	2.06E-13	4.39E-02	2.89E-06	8.96E-04	4.18E-02	1.67E-08	6.86E-06	7.20E-03	3.43E-05
PDLIM7	CP, CAC	+ -		-		+	+	+	-	+	+
		P-val		6.85E-04		1.19E-02	1.40E-03	9.14E-14	1.46E-08	5.02E-03	2.94E-04
LILRB4	CAC	+ -		-		+			-	+	
		P-val		4.32E-07		1.13E-03			4.84E-02	6.38E-03	
P2RX4	CAC	+ -		-	+	+		+	-	+	+
		P-val		8.79E-03	5.50E-10	2.36E-06		1.14E-03	3.05E-02	2.37E-02	8.01E-04
FAM168B	CAC	+ -		+				-			
		P-val		1.94E-09				2.94E-02			
ADM	CAC	+ -		-	+	+		+	-	+	
		P-val		1.04E-13	4.97E-06	1.11E-03		1.12E-08	9.21E-03	3.11E-02	
IARS	CAC	+ -	-	+		-		-	+	-	-
		P-val	2.60E-04	1.47E-11		3.52E-03		7.61E-06	2.78E-02	4.45E-02	1.01E-02

* Adjusting for demographic variables; sorted by most significant association with carotid plaque score or CAC; non-significant ($p \geq 0.05$) associations are left blank; three genes bolded had genome-wide FDR<0.05 for a direction of association with the majority of CVD risk factors similar to those expected based on the mRNA expression association with atherosclerosis and atherosclerosis associations with risk factors; LDL cholesterol was not associated with any atherosclerosis-associated gene expression.

Supplementary Table 8. Atherosclerosis-associated CpG methylation in monocytes associations with CVD risk factors*

CpG Illumina ID and nearby gene location	~ Carotid Plaque (CP) or CAC (FDR≤0.05)	Beta direction (+/-) or P-value	Age	Sex (male)	Race (Black)	Race (Hispanic)	Current smoker	BMI	HDL cholesterol	Hypertension	Diabetic
**cg06126421**	CP	+ -	-	-		+	-				
**Intergenic**		P-val	8.51E-12	2.67E-14		4.83E-08	1.47E-42				
**cg05575921**	CP	+ -		-	-	+	-	+			
**AHRR (Body)**		P-val		1.18E-11	2.26E-02	4.50E-02	1.59E-110	6.23E-03			
**cg21566642**	CP	+ -	-	-		+	-				
**Intergenic**		P-val	2.29E-02	1.61E-07		1.24E-03	1.74E-74				
cg05951221	CP	+ -		-			-				
Intergenic		P-val		2.47E-04			5.99E-71				
**cg06644428**	CP	+ -	+				-				
**Intergenic**		P-val	2.76E-02				4.07E-30				
**cg05119988**	CAC	+ -					-	-	+	-	-
**SC4MOL (5'UTR)**		P-val					2.64E-02	1.09E-04	1.09E-06	4.99E-03	1.27E-09
**cg19572487**	CP	+ -	-	-	+	+	-				
**RARA (5'UTR)**		P-val	7.24E-06	7.28E-06	5.59E-04	2.01E-05	1.20E-25				
**cg23661483**	CP, CAC	+ -	+		-	-	+				+
**ILVBL (Body)**		P-val	2.78E-09		1.30E-03	2.49E-07	3.60E-04				2.76E-03
**cg14753356**	CP	+ -	-	-		+	-				
**Intergenic**		P-val	5.08E-12	3.86E-06		1.09E-04	8.09E-32				
**cg09935388**	CP	+ -				+	-				
**GFI1 (Body)**		P-val				2.71E-03	1.84E-40				
**cg05546038**	CP	+ -		+	-		+				
**NOL3 (TSS1500)**		P-val		9.11E-08	4.96E-04		1.59E-08				
**cg10549018**	CAC	+ -	+	+	-		+				
**TLL2 (Body)**		P-val	2.09E-10	8.23E-06	2.92E-04		2.03E-03				
**cg21161138**	CP	+ -		-		+	-	+			
**AHRR (Body)**		P-val		4.20E-04		8.06E-04	3.01E-59	9.39E-03			
**cg15342087**	CP	+ -	-	-	-		-				
**Intergenic**		P-val	2.35E-06	1.74E-06	3.70E-05		3.21E-40				
**cg03295554**	CP	+ -				+	-	-		-	-
**ETS1 (Body)**		P-val				3.50E-02	1.54E-09	4.72E-03		5.11E-03	1.99E-03
**cg14026106**	CAC	+ -	-							-	
**MARCH8 (Body)**		P-val	1.66E-30							6.84E-03	
**cg24859433**	CP	+ -	-	-	-		-				-
**Intergenic**		P-val	1.26E-06	1.32E-10	1.63E-05		5.88E-34				3.66E-02
cg00889709	CAC	+ -	+	+	-		+	+	-	+	+
FAM110B (5'UTR)		P-val	1.36E-03	6.08E-04	4.11E-15		3.69E-02	7.34E-07	2.27E-03	2.20E-02	4.10E-07
**cg25953130**	CP	+ -	-	+	-	-	-		+		
**ARID5B (Body)**		P-val	3.33E-11	1.14E-06	1.10E-04	2.25E-02	7.19E-07		3.91E-03		
**cg00021275**	CP	+ -	+	+		+	+	+	-	+	+
**Intergenic**		P-val	2.96E-04	3.03E-13		2.50E-02	4.95E-05	8.31E-09	1.36E-03	1.14E-03	1.26E-06
**cg24247537**	CAC	+ -	+	+	+		+				+
**PTDSS2 (Body)**		P-val	1.89E-08	4.80E-05	9.08E-16		2.05E-05				9.70E-04
**cg21271420**	CP	+ -	-	-		+					
**Intergenic**		P-val	9.67E-27	7.86E-07		3.41E-04					
cg08867471	CP	+ -		-			-				
Intergenic		P-val		5.76E-06			6.96E-07				
**cg03636183**	CP	+ -		-	-		-	+			-
**F2RL3 (Body)**		P-val		2.42E-03	3.23E-09		8.48E-45	4.73E-02			1.39E-02
**cg01940273**	CP	+ -	-	-			-				
**Intergenic**		P-val	4.84E-06	2.82E-10			1.09E-62				
**cg04761231**	CP	+ -	-	+	+		-				
**RPL35 (Body)**		P-val	2.50E-04	1.46E-48	6.44E-07		1.18E-04				
**cg19266329**	CP	+ -	-	-	-		-				-
**Intergenic**		P-val	1.10E-19	1.72E-08	1.60E-03		1.45E-06				1.80E-05
**cg15501219**	CP	+ -	+	-			+	+			+
**Intergenic**		P-val	9.82E-05	1.17E-111			3.69E-04	7.31E-05			5.80E-06
cg24574691	CP	+ -		+	-	-				+	

**CpG Illumina ID and nearby gene location**	**~ Carotid Plaque (CP) or CAC (FDR≤0.05)**	**Beta direction (+/-) or P-value**	**Age**	**Sex (male)**	**Race (Black)**	**Race (Hispanic)**	**Current smoker**	**BMI**	**HDL cholesterol**	**Hypertension**	**Diabetic**
Intergenic		P-val		1.21E-02	3.91E-06	2.11E-02				5.72E-03	
**cg00834988**<br>**AUTS2 (Body)**	CP	+ -<br>P-val		+<br>3.87E-02			+<br>2.38E-10			+<br>2.05E-02	+<br>2.66E-03
**cg13200854**<br>**MMRN1 (Body)**	CP	+ -<br>P-val	-<br>4.70E-14	-<br>6.14E-03			-<br>8.55E-03			-<br>1.54E-02	
**cg08958747**<br>**RAB26 (Body)**	CP	+ -<br>P-val	-<br>7.46E-29	+<br>3.70E-07	+<br>2.56E-05	+<br>1.79E-02	-<br>1.15E-03				
**cg01763719**<br>**Intergenic**	CP	+ -<br>P-val					+<br>1.26E-06	+<br>1.24E-02			

* Adjusting for demographic variables; sorted by most significant association with carotid plaque score or CAC; non-significant ($p \geq 0.05$) associations are left blank**;** 29 CpGs bolded had genome-wide FDR<0.05 for a direction of association with the majority of CVD risk factors similar to those expected based on the methylation association with atherosclerosis and atherosclerosis associations with risk factors; LDL cholesterol was not associated with any atherosclerosis-associated CpG methylation.

Supplementary Table 9. siRNA Knockdown of ARID5B Alters Immune/inflammatory Response and Lipid Metabolism Genes.

Terms		Down-regulated genes	Up-regulated genes
**Bio-Functions**	FDR		
**Inflammatory response**	1.10E-06	ABCD1, ABHD12, ABR, ADA, ADORA2B, ADORA3, ADRBK1, AGT, AKT1, ALOX5, APOE, ATF3, ATM, AXL, BCL6, C3AR1, C5AR1, CASP1, CASP4, CCL14, CCL3, CCL3L1, CCL4, CCRL2, CD14, **CD1D**, CD36, CD4, **CD74**, CD9, CDC42, CIRBP, CKLF, CLCF1, CLCN7, CORO1A, CSF1R, CST3, CTSB, CTSG, CXCL2, CYBB, CYSLTR1, DUSP10, EDN1, EGR1, EIF2AK1, ELANE, ELMO1, ENG, EPAS1, ERN1, F11R, FABP4, FCGR1A, FCGR2A, **FCGRT**, FGR, FOXF1, FPR1, GNA13, GNAI2, GRN, HAVCR2, HDAC4, HEBP1, **HLA-DMA, HLA-DRA, HLA-DRB1, HLA-DRB5, HLA-DRB6,** HMOX1, HPSE, **IFNB1, IL1A**, INPP5D, **IRF3**, ITGB1, KLF4, LAT, LGALS9, LILRB3, LIPA, LTB4R, LY96, MCL1, MDK, MPP1, MR1, MYO1F, MYO9B, NCKAP1L, NFIL3, NFKBIZ, NLRP3, NR4A2, OSTM1, PARP4, PDE4B, PECAM1, PIK3CB, PLA2G7, PLAU, PLCG2, PON2, PPARG, PPT1, PRDM1, PRKCA, PRNP, PROCR, PRTN3, PSEN2, **PSMB8, PSMB9**, PTGER2, PTGER4, PTGS1, PTGS2, PTPN11, PTPRO, PYCARD, RAC2, RGS1, RHOB, RIPK3, RNASE2, RPL13A, SERPINF1, SIGIRR, SLC11A1, SLC1A5, SMAD7, SPHK1, **STAT1, STAT2**, STAT6, STK39, SYK, TBXAS1, TGM2, TLR4, **TNF**, TNFRSF14, TNFSF14, TREM2, TYROBP, ULBP1, WAS, ZFP36	ABCF1, AHR, ALOX5AP, ANGPTL4, AQP9, BID, BRD2, CCND1, CD40, CD44, CEBPD, CXCL6, E2F2, FEM1A, FYN, GJA1, HDAC9, **HLA-H**, HNRNPA0, ICAM1, IDO1, IFNAR1, IKBKB, **IL10**, IL10RA, IL18RAP, IL23A, JMJD6, LILRB3, LRIG2, LYN, LYST, MAPK7, MGLL, MIF, NCL, NFE2L1, NFKBIB, NFX1, NOD2, NOTCH1, OLR1, ORM1, P2RY6, PGLYRP2, PIK3CD, PPM1D, PRKCD, PTX3, S100A12, S100A8, S100A9, SBNO2, SFRP1, SIRPA, SLC33A1, SLC7A2, SMAD3, SRC, STAT4, STAT5A, TICAM2, TNFAIP8L2, TNFRSF1A, TNFRSF1B, TNFRSF4, TNFRSF9, TNIP1, TRADD, TRAF3IP2, TRNT1, TSC1, VAV3
**Chemotaxis of leukocytes**	1.38E-02	ADORA3, ALOX5, APOE, C3AR1, C5AR1, CCL14, CCL3, CCL3L1, CCL4, CCRL2, CD4, CD74, CD9, CDC42, CKLF, CORO1A, CSF1R, CST3, CTSG, CXCL2, CYBB, CYSLTR1, EDN1, ELANE, ELMO1, FCGR2A, FGR, FPR1, GNA13, GNAI2, GRN, HEBP1, HPSE, IFNB1, IL1A, INPP5D, LAT, LGALS9, LILRB3, LTB4R, MDK, MPP1, MYO1F, MYO9B, NCKAP1L, PDE4B, PIK3CB, PLA2G7, PLAU, PLCG2, PON2, PPARG, PRKCA, PRNP, PRTN3, PTPN11, PTPRO, RAC2, RGS1, RHOB, RNASE2, RPL13A, SPHK1, STAT6, SYK, TLR4, TNF, TNFRSF14, TNFSF14, WAS	AQP9, CCND1, CD40, CXCL6, FYN, ICAM1, IKBKB, IL10, IL23A, JMJD6, LYN, LYST, MIF, NOD2, PIK3CD, S100A12, S100A8, S100A9, SMAD3, TNFRSF1A, TRAF3IP2, TSC1, VAV3
**Migration of phagocytes**	1.69E-03	ADAM15, AGT, APOE, BMPR2, CCL3, CCL4, CD209, CD74, CD86, CDC42, CSF1R, CTSG, CTSZ, CXCL2, CYBB, DOCK8, ENG, EPAS1, F11R, FGR, FLNA, FPR1, HCLS1, IFNB1, INPP5D, ITGAX, ITGB1, MDK, MYO9B, NAB2, NARS, NLRP3, PDE4B, PECAM1, PLAU, PLCB2, PPARG, PRNP, PTGDS, PTGS2, RAC2, RHOB, RTN4, SEMA4D, SERPINF1, TIMP2, TIMP3, TLN1, TLR4, TNF, TREM2, TYROBP, VCAN, WAS	ABL1, CCND1, CD40, CD44, CD58, CD80, CD99, CXCL6, ICAM1, ICAM2, IL10, IL10RA, MIF, P2RY6, PIK3CD, PPM1D, PVR, QPCT, S100A8, S100A9, SH2B3, SIRPA, TNFAIP8L2, TNFRSF1A, TNFRSF9, VAV3
**Phagocytosis of blood cells**	3.00E-03	BIN1, BTK, CD14, CD36, CD93, CDC42, CERK, CLCN3, CORO1A, CORO1C, CYP2S1, DEF6, EIF2AK1, **FCGR1A**, **FCGR1B**, **FCGR1C**, FCGR2A, FGR, FPR1, GAB2, **GAS6**, GLRX, HMOX1, INPP5D, ITGAX, MERTK, PIK3CB, PLAU, RAC2, STIM1, SYK, TGM2, TLR4, TNF, TREM2, UCP2, WAS	CD44, FYN, IFNAR1, IRF8, JMJD6, LYN, MIF, NOD2, PTX3, S100A9, SIRPA, SRC
**Apopotosis of leukocytes**	6.79E-04	ABCG1, ADA, ADORA3, AKT1, ANPEP, APOE, ASAH1, ATF3, ATM, BTK, C3AR1, CASP1, CASP4, CAT, CCL3, CCL4, CD14, CD4, CD9, CDC42, CORO1A, CXCL2, CYBB, DOCK8, EGR1, EGR3, ETS2, FCGR1A, FGL2, FOSL1, GADD45B, GPR132, HAVCR2, IFNB1, IL1A, INPP5D, IRF3, IRF4, IRS2, ITGB1, ITPR1, KLF4, LGALS9, MAP2K5, MAP4K1, MCL1, MERTK, MYC, NFATC1, NR4A1, NR4A3, PECAM1, PKN1, PLCG2, PPARG, PRKCA, PRTN3, PTGER4, RAC2, RCAN1, RIPK3, SERPINF1, SLC39A10, SMAD7, SPHK1, ST14, STAT1, STAT6, TLR4, TMEM109, TNF, TNFRSF21, TNFSF14, TOP2A, WAS	ABL1, AHR, BCL11A, BCL2, BCL2L1, BCL3, BID, BMF, CASP2, CCDC86, CD40, CD44, CD70, CD80, CDKN1B, CREB1, DLL4, DNAJA3, E2F2, FBXW7, FYN, ICAM1, IDO1, IKBKB, IKBKE, IL10, IL7R, IRF8, LILRB1, LYN, MIF, MYB, NFKBIB, NOTCH1, OGT, PERP, PIK3CD, PIM1, POLR3E, PPM1D, PRELID1, PRKCD, RCAN2, S100A8, SERPINB9, SMAD3, SOD2, ST6GAL1, STAT5A, TICAM2, TNFAIP8L2, TNFRSF1A, TNFRSF1B, TNFRSF4, TNFRSF9, TNFSF10, TNFSF13B, TRAF3IP2, TSC1, TSC22D3, VAV3
**Synthesis of lipid**	2.10E-04	ABCG1, ACSL3, ACSS2, ADA, ADORA2B, ADRBK1, AGPAT1, AGPAT2, AGT, AKT1, ALDH1A2, ALOX5, ANPEP, APOC1, APOC2, APOE, ARV1, ASAH1, ATF3, B3GNT5, BMP2, BMPR2, BRE, C5AR1, CASP1, CD14, CD209, CD36, CD4, CD74, CD9, CDC42, CDIPT, CERK, CHKB, CLN3, CYP1B1, DAB2, DGAT1, DHRS9, DOLK, EDN1, EEF1A1, EGR1, ERLIN2, FABP5, FAR1, FCGR1A, FCGR2A, FGR, FOSL1, FOXA1, FOXO4, FPR1, GBA, GBGT1, GDF15, GNPAT, **HMGCS1,** HMOX1, HOXA10, IL1A, INPP5D, INPPL1, INSIG1, ITGB1, LTA4H, LTB4R, LTC4S, MAPK3, ME2, MGST2, MITF, MKNK1, MYC, NPC2, NR4A1, NR4A2, NR4A3, NSMAF, PDHB, PDSS1, PDSS2, PIGB, PIGP, PIGY, PIK3C2A, PIK3CB, PISD, PITPNM1, PLAU, PLCG2, PMVK, PNPLA2, PNPLA6, PON2, POR, PPARG, PRKAA1, PRKAG2, PRKCA, PRNP, PTGDS, PTGER2, PTGS1, PTGS2, PTPN11, RBP1, RGS2, RXRA, **SC5DL**, **SCD**, SH3KBP1, SIGIRR, SLC9A3R1, SPHK1, SPTLC1, ST3GAL5, SYK, TBXAS1, TLR4, TNF, TRERF1, TRIB3	ACAT2, ACSL5, AHR, AKR1B1, ALOX5AP, ATP5J, BCL2, BCL2L1, BMP6, BNIP3, BRD2, C1QTNF1, CD40, CD82, CES1, CREB1, CYP7B1, DEGS1, FAF2, FYN, IGFBP4, IL10, IL18RAP, INSIG2, LYN, MAP3K1, MAP3K8, MCAT, ME1, MIF, NR5A2, OLR1, OSBP, PEX11B, PGS1, PIAS1, PIGV, PIK3C2B, PIK3CD, PPAT, PRDX2, PRKCD, PTPMT1, PTX3, RDH10, SLC1A3, SLC35A2, SMAD3, SMAD4, STAT5A, TNFRSF1A, TNFRSF1B, TNFSF10, UGCG
**Canonical pathways**	FDR		
**Leukocyte Extravasation Signaling**	8.31E-03	ACTB, ACTG1, ACTN1, ACTN4, ARHGAP4, ARHGAP5, ARHGAP9, ATM, BTK, CDC42, CLDN1, CLDN23, CTNNA1, CYBB, F11R, GNAI2, ITGB1, MAP3K4, MAPK13, MMP10, NCF2, NCF4, PECAM1, PIK3C2A, PIK3CB, PIK3R2, PLCG2, PRKCA, PRKCH, PTPN11, RAC2, SIPA1, TIMP2, TIMP3, VCL, WAS	ABL1, CD44, CD99, CRKL, ICAM1, PIK3C2B, PIK3CD, PRKCD, PTK2, RASGRP1, RDX, SRC, VAV3

Enrichment from IPA ingenuity, including genes significantly (FDR<0.05) altered after siARID5B in in LPS stimulated human THP1-monocytes, compared to a background of all genes with detectable expression

Supplementary Methods

RNA sequencing

Total RNA samples were enriched for mRNA, by depleting rRNA using the MICROBExpress kit from Ambion and following the manufacturer's instructions. Poly(A) mRNA was enriched, and Illumina compatible, strand-specific libraries were constructed using Illumina's TruSeq Stranded mRNA HT Sample Prep Kit (Illumina, RS-122-2103). 1 ug of total RNA with RIN ≥ 8.0 was converted into a library of stranded template molecules suitable for subsequent cluster generation and sequencing by Illumina HiSeq. The libraries generated were validated using Agilent 2100 Bioanalyzer and quantitated using Quant-iT dsDNA HS Kit (Invitrogen) and qPCR. Six individually indexed cDNA libraries were pooled and sequenced on Illumina HiSeq, resulting in an average of close to 30 million reads per sample. Libraries were clustered onto flow cells using Illumina's TruSeq PE Cluster Kit v3 (PE-401-3001) and sequenced 2X100 cycles using TruSeq SBS Kit -HS (FC-401-3001) on an Illumina HiSeqTM 2500. A total of 64 lanes were run to generate approximately 30 million 2 x 101 Paired End reads per sample. The Illumina HiSeq Control Software (HCS v2.0.12) with Real Time Analysis (RTA v1.3.61) was used to provide the management and execution of the HiSeq 2500.

Illumina sequencing runs were processed to de-multiplex samples and generate FastQ files using the Illumina provided *configureBclToFastq.pl* script to automate running CASAVA 1.8.4 using default parameters for removal of sequencing reads failing the chastity filter (yes) and mismatches in the barcode read (0). Following generation of FastQ files, reads were trimmed to remove poor quality reads (or read tails) using *Btrim* (5 base sliding window average with $Q > 15$) [1] and then trimmed to remove any adaptor sequence present in the reads using custom perl scripts (trim sequences containing 11 base tag of adaptor, final length >40 bases). The *Ensembl* GRCh37 *Homo Sapiens* reference file, annotations and Bowtie2 indexes were downloaded from the *igenomes.com* website (10-Apr-2013) for mapping of the sequencing reads to the genome and read counting. *Bowtie2 (2.1.0)* and *TopHat2 (2.0.8)* were used to map the sequencing reads to the genome using a mate-inner-distance of 100 bp and '*firststrand*' options [2, 3]. Following alignment, *bam* files were merged using the *samtools* (0.1.19) merge function [4], and read counts per gene were obtained using *HTSeq* (0.5.4p3) (http://www-huber.embl.de/users/anders/HTSeq /doc/overview.html). The '*intersection-strict*' overlap resolution mode and '*stranded reverse*' options were used in *HTSeq*.

Data pre-processing and QC analyses were performed in *R* (http://www.r-project.org/) using *Bioconductor* (http://www.bioconductor.org/) packages. The transcript-based raw count data files for each sample from *TopHat2* were combined into a count matrix with 56,303 features (rows) and 374 MESA samples (columns). The median total count per sample was 28.8 million. Reads denoted by *TopHat2* as "no_feature","ambiguous", "too_low_aQual", "not_aligned", "alignment_not_unique" were removed. Counts were converted to Counts Per Million (CPM) using the *cpm* function of the *edgeR* package [5] , and all features with CPM ≤ 0.25 in ≥90% of the 374 MESA samples were removed. Features assigned to the mitochondrial genome were removed as well. Using the *biomaRt* package and querying the *Ensembl BioMart* database, *Entrez Gene ID*s, Gene Symbols, genome coordinates, gene length and percent GC content were obtained for 12,585 features which had a corresponding *Entrez ID or* Illumina HumanHT-12 v4 probe ID. To be able to continue to use the flexible and computationally efficient linear modeling functions in *R,* we transformed the raw count data to log counts per million (y = logCPM) as recommended by Law et al (2013)[6]:

$$y_{gs} = log_2\left(\frac{c_{gs} + 0.5}{T_s + 1} \cdot 10^6\right)$$

where c_{gs} is the raw count of gene transcript g in sample s, and T_s is the normalized total count of sample s, using the Trimmed Mean of M-values (TMM) normalization method [7] as implemented in the *calcNormFactors* function in the *edgeR Bioconductor* package [5]. We either performed only this TMM normalization, or we applied quantile normalization (QN) to the logCPM values. Because the logCPM values' variance tends to decrease with increasing count for smaller counts, we used the voom function of the *limma* package [8] to estimate the mean-variance trend non-parametrically and to predict the residual variance of each individual observation for each gene. Then we incorporated the inverse residual variances into the linear modeling (*lm*) as weights in a standard manner. For the logCPM data, we imposed the same low variance filter that we had used for the microarray data, removing another 192 features with the lowest variance and retaining 12,380 features for analysis. We then performed weighted linear model analyses with the otherwise exact same models as for the microarray data.

Bisulfite treatment of genomic DNA and pyrosequencing

Genomic DNA from monocytes was bisulfite modified using the EZ DNA Methylation™ Kit (Zymo Research Co., Irvine, CA) according to the manufacturer's protocol for the Infinium methylation assay. Primers (F1-Biotinated 5'-GAAAATAGGAAATGTTTAATTGTG-3', R1 5'-TCACTATACTCCTAACAACCAACC-3') for pyrosequencing assays of *ARID5B*-cg25953130 were designed using PSQ assay design software version 1.0.6 (Biotage, Uppsala,

Sweden). PCR was performed with the PyroMark PCR Master Mix (Qiagen Inc.). Pyrosequencing was conducted using PyroMark Gold Q98 Reagents (Qiagen Inc.). Methylation values for each CpG site were calculated using Pyro Q-CpG software 1.0.9 (Biotage).

Supplementary References

1. Kong,Y. Btrim: a fast, lightweight adapter and quality trimming program for next-generation sequencing technologies. *Genomics* **98**, 152-153 (2011).

2. Langmead,B., Trapnell,C., Pop,M., & Salzberg,S.L. Ultrafast and memory-efficient alignment of short DNA sequences to the human genome. *Genome Biol.* **10**, R25 (2009).

3. Trapnell,C., Pachter,L., & Salzberg,S.L. TopHat: discovering splice junctions with RNA-Seq. *Bioinformatics.* **25**, 1105-1111 (2009).

4. Li,H. *et al.* The Sequence Alignment/Map format and SAMtools. *Bioinformatics.* **25**, 2078-2079 (2009).

5. Robinson,M.D., McCarthy,D.J., & Smyth,G.K. edgeR: a Bioconductor package for differential expression analysis of digital gene expression data. *Bioinformatics.* **26**, 139-140 (2010).

6. Law,C.W., Chen,Y., Shi,W., & Smyth,G.K. Voom! Precision weights unlock linear model analysis tools for RNA-seq read counts. Preprint available at http://www.statsci.org/smyth/pubs/ . 2013.
Ref Type: Generic

7. Robinson,M.D. & Oshlack,A. A scaling normalization method for differential expression analysis of RNA-seq data. *Genome Biol.* **11**, R25 (2010).

8. Smyth,G.K. Limma: linear models for microarray data in *Bioinformatics and Computational Biology Solutions using R and Bioconductor* (eds. Gentleman,R., Carey,V., Dudoit,S., Irizarry,R. & Huber,W.) 397-420 (Springer, New York, 2005).
